# Use of adenoviral E1A protein to analyze K18 promoter deregulation in colon carcinoma cells discloses a role for CtBP1 and BRCA1

**DOI:** 10.1186/1471-2199-6-8

**Published:** 2005-04-14

**Authors:** Cécile Delouis, Philippe Prochasson, Madeleine Laithier, Olivier Brison

**Affiliations:** 1 Laboratoire de Génétique Oncologique, UMR 8125 CNRS, Institut Gustave Roussy, 39 rue Camille Desmoulins, 94805 Villejuif, France; 2 PP: Stowers Institute, 1000 E 50^th ^street, Kansas City, MO 64110, USA; OB: UMR 7147, Institut Curie, 26 rue d'Ulm,75248 Paris cedex 05, France

## Abstract

**Background:**

The promoter of the keratin 18 (K18) gene is 5- to 10-fold more active in tumorigenic (T-type) cell clones derived from the SW613-S human colon carcinoma cell line than in non-tumorigenic (NT-type) clones. We have reported previously that the mechanism responsible for this differential activity is acting on the minimal K18 promoter (TATA box and initiation site). This mechanism does not require the binding of a factor to a specific site on the DNA but involves the acetylation of a non-histone substrate. To get further insight into this mechanism, we investigated the effect of the adenovirus E1A protein on the activity of the K18 promoter, both in T and NT cells.

**Results:**

Wild type adenovirus E1A protein and C-terminal deletion mutants inhibit the K18 promoter, specifically in T-type cells. The domain responsible for this inhibitory effect is located in the 12–25 region of the viral protein. E1A mutants that have lost this region but retain the PLDLS motif (the C-terminal binding site for CtBP1) stimulate the K18 promoter, specifically in NT cells. The inhibitory or stimulatory effects of the different E1A mutants are not dependent on a particular sequence of the promoter. An E1A N-terminal deletion mutant carrying point mutations in the PLDLS motif cannot stimulate the K18 promoter. CtBP1 interacts with CtIP, which is a known partner of BRCA1, itself a component of the RNA polymerase II holoenzyme. The stimulatory effect of two BRCA1 mutants, specifically in NT cells, implicates a tripartite BRCA1-CtIP-CtBP1 complex in the regulation of the K18 promoter.

**Conclusion:**

Since we have shown previously that the K18 promoter is stimulated by deacetylase inhibitors, specifically in NT cells, we conclude that the activity of the promoter is repressed in NT cells by a mechanism involving the recruitment, by a BRCA1/CtIP complex, of CtBP1 and associated deacetylases to the preinitiation complex. We propose a model depicting the mechanism responsible for the differential activity of the K18 promoter between T and NT cells of the SW613-S cell line.

## Background

The early region 1A (E1A) of adenoviruses encodes two main proteins (243 and 289 aa-long in human adenovirus 2) which are translated from alternatively spliced mRNAs (12S and 13S, respectively). The two proteins have identical N- and C-terminal regions but the larger one (E1A-13S) has an additional domain (46 aa-long in adenovirus 2) located in the central part of the protein. Four regions that are conserved between several human adenoviruses were named CR1, CR2, CR3 and CR4. In adenovirus 2, CR3 almost coincides with the 46 aa-long additional domain present in the E1A-13S isoform. The E1A-12S and E1A-13S proteins are required to activate the transcription of other viral genes. In addition, these proteins interact with multiple cellular proteins to reprogram the expression of many cellular genes in infected cells (reviewed in [1-3]). This is necessary for the virus to replicate its DNA and complete a productive cycle. The E1A proteins were also found to be oncogenic in primary rodent cells. This is a consequence of their ability to interact with key cellular factors, including regulators of the cell cycle such as the pocket proteins (Rb, p107, p130), the related p300 and CBP coactivators of transcription or the cyclin-dependent kinase inhibitors p21^Cip1/Waf1 ^and p27^Kip1^. Among the other cellular factors known to interact with E1A are the TRRAP, p400 and CtBP1 proteins. CtBP1 is a transcriptional corepressor. Several transcriptional repressors recruit CtBP1 to promoters through a conserved PXDLS motif. CtBP1 represses transcription either directly or by subsequent recruitment of histone deacetylases (HDACs) [4]. E1A proteins interact with CtBP1 by a PLDLS motif located at the C-terminus of the viral proteins [5]. A PLDLS motif is also present in the binding site of the cellular CtIP protein, another interacting partner of CtBP1 [4,6]. The function of CtIP in the cell is unknown but it was found to interact with the BRCT repeats of the BRCA1 tumor suppressor protein [7,8]. CtIP binds to BRCA1 through a site distinct from the PLDLS motif [9]. Thus, CtIP can act as an adapter protein between BRCA1 and CtBP1.

We are interested in the mechanisms involved in transcriptional deregulation of gene expression in the cells of the SW613-S cell line derived from a human colon carcinoma [10]. Analysis of cellular clones isolated from this cell line indicated that it is heterogeneous and composed of a mixture of two main cell types (named here T and NT). T-type cells have a high level of amplification and expression of the c-*MYC *gene, whose additional copies are located on extrachromosomal elements (double minute chromosomes). NT-type cells present a low level of amplification of the oncogene and the supernumerary copies are integrated into chromosomal DNA [11-14]. T and NT cells markedly differ by several phenotypic traits such as their tumorigenic potential in nude mice, capability to grow in serum-free medium, sensitivity to the induction of apoptosis and cellular morphology [12,15,16]. Genes overexpressed in T cells, as compared to NT cells, have been identified [12,17-20]. Among them, we chose the keratin 18 (K18) gene to investigate the mechanism responsible for its overexpression in T-type cells. We previously reported that this high level of expression is mainly due to an increase in transcriptional rate [17] and that, in transient expression assays, the K18 promoter is much more active in T cells than in NT cells [21]. The mechanism responsible for this higher activity is acting on the minimal K18 promoter (TATA box and initiation site) and does not involve the binding of a factor to a specific site on the DNA [22]. We also found that an acetylation mechanism acting on a non-histone substrate is driving the high activity of the promoter in T cells [23]. In order to get further insight into this mechanism, we now investigated the effect of the adenovirus E1A protein on the activity of the K18 promoter, both in T and NT cells. This protein has been widely used as a powerful tool to identify and/or study important cellular regulatory proteins, in particular factors involved in acetylation mechanisms (p300/CBP, PCAF, TRRAP) [24]. Using a series of E1A mutant proteins, we uncovered a role for the CtBP1 and BRCA1 proteins in the functioning of the K18 promoter in SW613-S cells.

## Results

### Wild type E1A and E1A mutants differentially inhibit or stimulate the minimal K18 promoter

**Figure 1:**
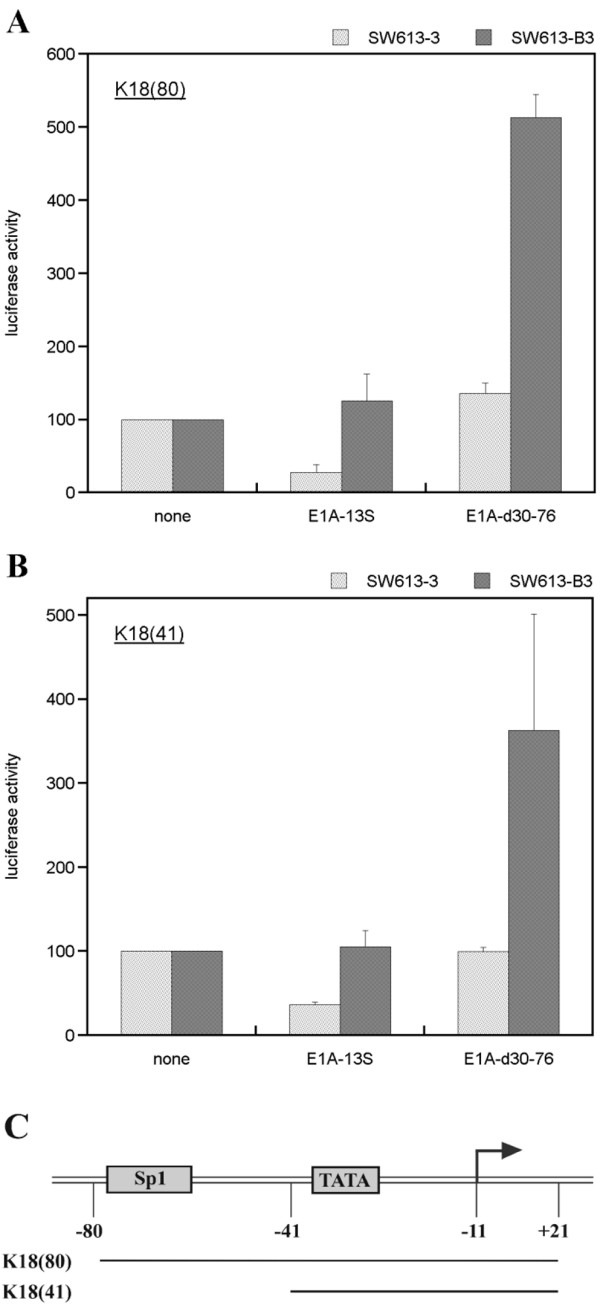
**Effect of E1A-13S or E1A-d30-76 proteins on the activity of the K18 promoter**. SW613-3 (T) or SW613-B3 (NT) cells were transfected with plasmid pK18(80)luc **(A) **or plasmid pK18(41)luc **(B) **alone (none) or in the presence of plasmid pE1A-13S or pE1A-d30-76, as indicated. Luciferase activities are expressed relative to that obtained with the reporter plasmid alone for each cell clone. Error bars represent the standard deviation of the mean of two experiments. **(C) **Structure of the promoter of the human K18 gene. The binding site of the Sp1 transcription factor, the initiation site (arrow at position -11; we have shown that the initiation is located 11 bp upstream of previously published +1 position [22]) and the TATA box are indicated. Numbers indicate positions in nucleotides. K18(80) and K18(41), the two forms of the promoter inserted into luciferase expression vectors are shown.

We previously found that the adenoviral E1A-12S protein has an inhibitory effect on the K18 promoter, specifically in T-type cells of the SW613-S cell line. In contrast, a mutant form of the protein, lacking the CR1 domain (E1A-12S-ΔCR1), has lost this inhibitory effect but gained a stimulatory activity on the K18 promoter, specifically in NT cells [23]. All the E1A mutants that we planned to use in this work were derived from the E1A-13S isoform. Therefore, the effect of the E1A-13S protein and of the E1A-d30-76 mutant protein (equivalent to E1A-12S-ΔCR1) on the activity of the K18(80) promoter (-80 to +21) was assayed. As shown in figure 1A, the E1A-13S protein has also a strong inhibitory effect on the promoter activity in T cells whereas it has no significant effect in NT cells. In contrast, the E1A-d30-76 mutant behaves like the E1A-12S-ΔCR1 protein and stimulates the activity of the promoter, specifically in NT cells.

**Figure 2:**
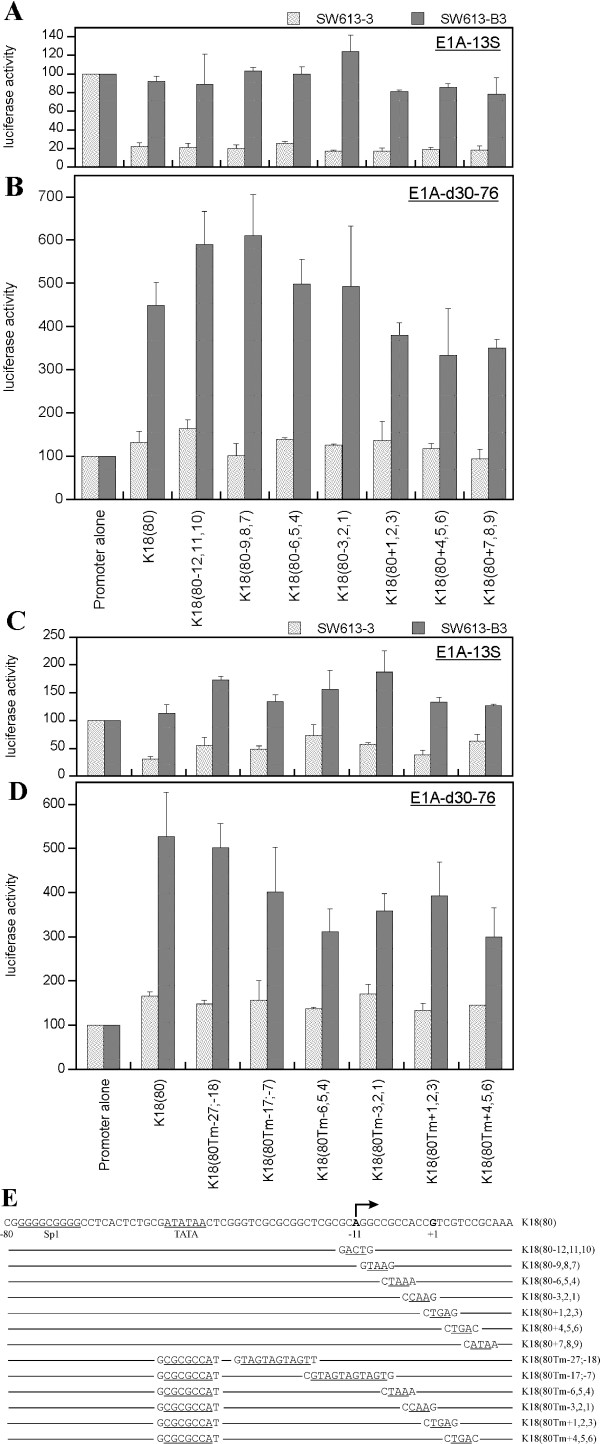
**Effect of E1A-13S or E1A-d30-76 proteins on the activity of mutated versions of the K18 promoter. **SW613-3 (T) or SW613-B3 (NT) cells were transfected with each of the reporter plasmids carrying the indicated mutated form of the K18(80) promoter, alone or in the presence of plasmid pE1A-13S **(A **and **C) **or plasmid pE1A-d30-76 **(B **and **D)**. Luciferase activities are expressed relative to that obtained with the promoter construct alone in each cell line. Since the activities of all promoter constructs alone were adjusted to 100, they are all represented by a single pair of columns in each panel. The activity of these constructs relative to that of the wild type K18(80) promoter has been reported previously [22]. The structure of the various mutant forms of the K18(80) promoter is schematized in **E**. Error bars represent the standard deviation of the mean of duplicate experimental points.

The promoter of the K18 gene comprises a TATA box and an initiation site (minimal promoter) flanked by an Sp1 binding site (Fig. 1C). The minimal K18(41) promoter (-41 to +21) has a low but differential activity between T and NT cells. The flanking Sp1 binding site is essential for a high activity of the promoter but not for its differential behavior [21]. The effect of the E1A-13S protein and of the E1A-d30-76 mutant was also assayed on the minimal K18(41) promoter (Fig. 1B). The former inhibits the activity of the minimal promoter, specifically in T cells, and the latter stimulates it only in NT cells, indicating that the Sp1 binding site is not essential for these effects. By mutational analysis of the K18 minimal promoter, we have previously shown that the factor(s) responsible for its differential activity between T and NT cells does not bind to a specific DNA sequence [22]. Using the same mutant constructs of the K18 promoter (Fig. 2E), we investigated whether the inhibitory or stimulatory effects of the E1A-13S and E1A-d30-76 proteins were also sequence-independent. As shown in figure 2A–D, all the promoter constructs are inhibited by E1A-13S, specifically in T cells, and are stimulated by the E1A-d30-76 mutant, only in NT cells. These results suggest that, as for the differential activity of the promoter, the effect of these viral proteins is not mediated by a factor which binds to a specific site on the promoter but rather through alterations of protein-protein interactions within the preinitiation complex.

### An N-terminal domain of E1A is involved in the inhibitory effect on the K18 promoter

**Figure 3:**
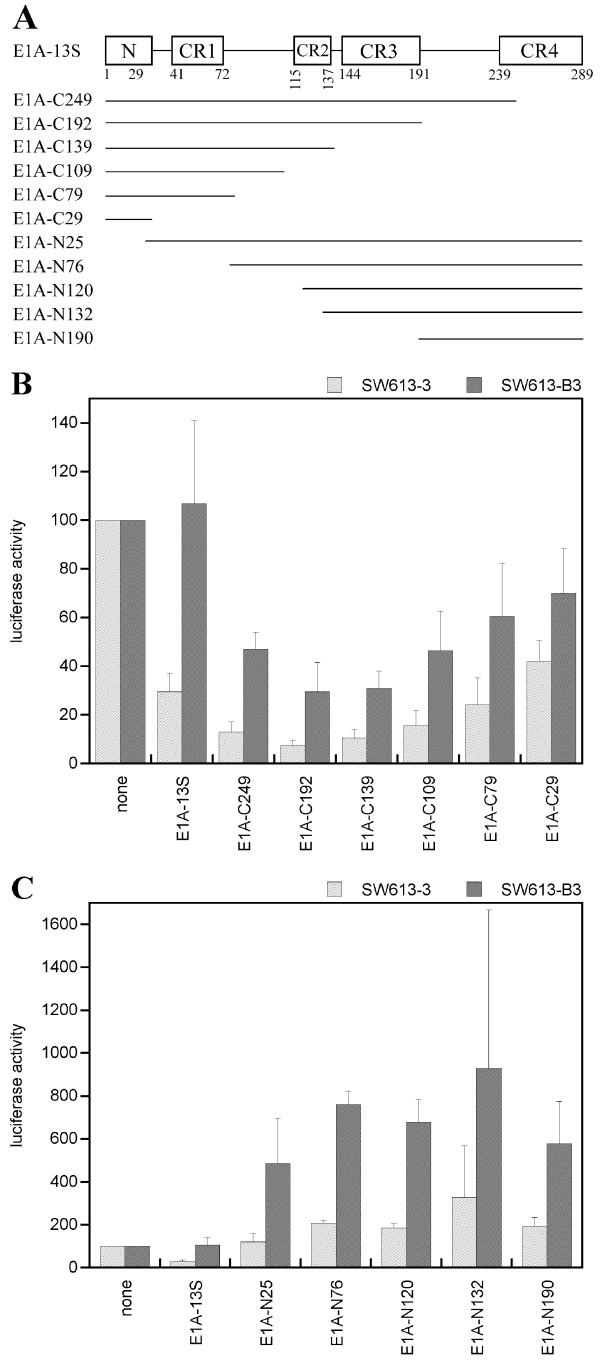
**Effect of C-terminal and N-terminal deletion mutants of the E1A protein on the activity of the K18 promoter. (A) **Schematic representation of the structure of the E1A-13S protein and of the deletion mutants. Know domains of E1A-13S (CR1, CR2, CR3 and CR4 and the N-terminal region) are shown as boxes. Numbers indicate positions in aa. **(B **and **C) **SW613-3 (T) or SW613-B3 (NT) cells were transfected with plasmid pK18(80)luc alone (none) or in the presence of the indicated E1A constructs. Luciferase activities are expressed relative to that obtained with the pK18(80)luc plasmid alone. Error bars represent the standard deviation of the mean of at least two experiments.

**Figure 4:**
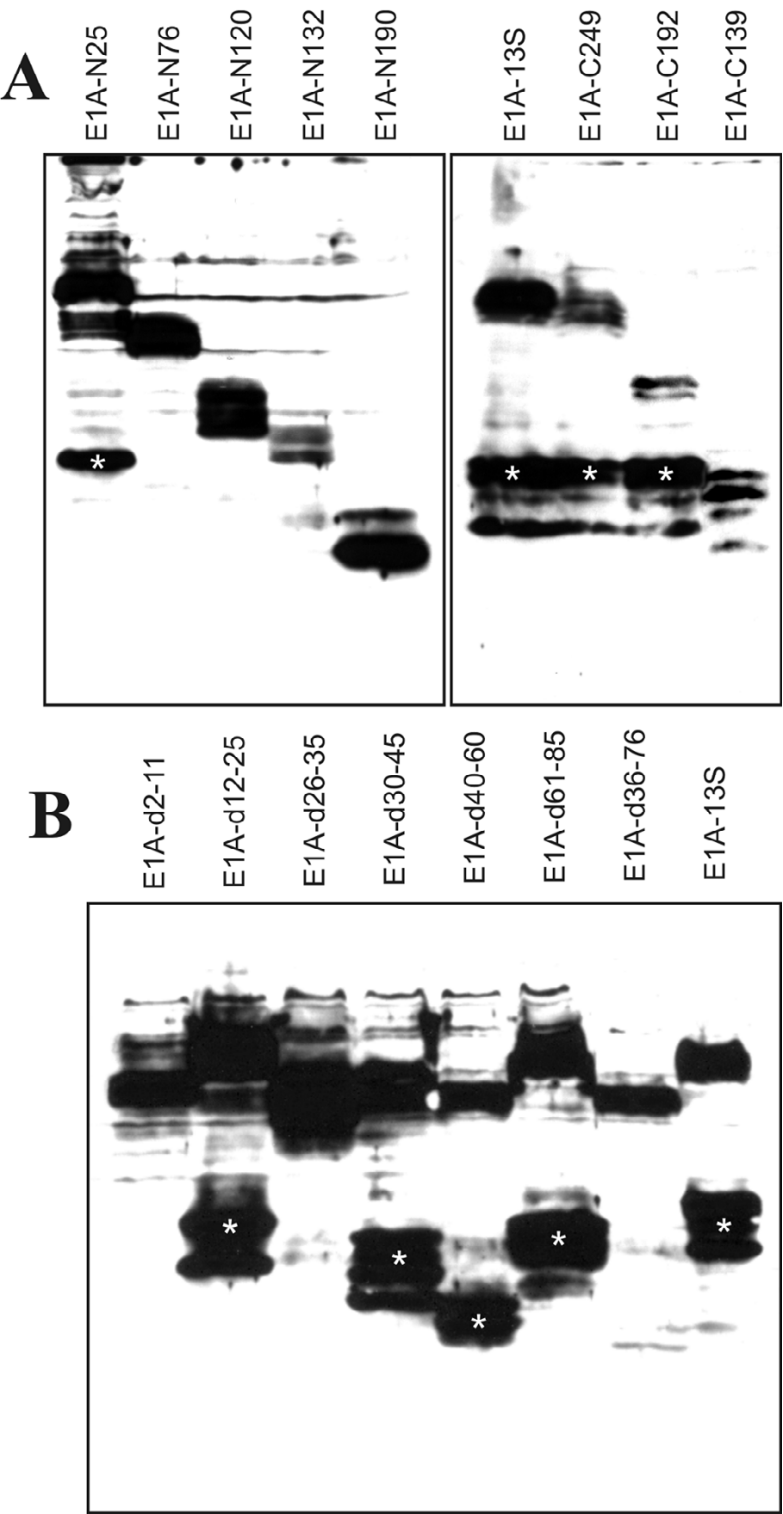
**Expression level of the E1A constructs**. SW613-3 cells were transfected with plasmid constructs coding for the indicated wild-type or mutant E1A proteins. Cellular extracts were analyzed by Western blotting with an anti-E1A antibody. **(A) **N- and C-terminal deletion mutants. The E1A-C29, -C79 and -C109 polypeptides cannot be detected with this antibody. **(B) **Internal deletion mutants. Note that E1A and some of its mutants are very sensitive to proteolysis (white asterisks) in spite of the presence of protease inhibitors. Similar results were obtained in SW613-B3 cells (not shown).

In order to identify the region(s) of the E1A protein involved in the inhibition of the K18 promoter in T-type cells, we first used a series of N- or C-terminal deletion mutants (Fig. 3A). All the C-terminal deletion mutants have a comparable inhibitory effect on the activity of the K18 promoter in T-type cells (Fig. 3B). Mutant E1A-C29, which comprises only the N-terminal 29 aa, seems less efficient at inhibiting the promoter. Further careful comparative analysis indicated that the inhibitory potency of this mutant is about 60% of that of the E1A-13S protein (data not shown). Some of the mutants also acquired an inhibitory potential in NT cells but this point was not explored further. None of the C-terminal deletion mutants display a stimulatory effect on the promoter in NT cells. The localization of the inhibitory domain in the first 29 aa of E1A was confirmed by the analysis of N-terminal deletion mutants (Fig. 3C). Mutant E1A-N25, deleted of the first 25 amino acids, has lost the inhibitory potential in T cells but has acquired the ability to stimulate the promoter in NT cells. This was also the case for all the other N-terminal deletion mutants. The expression level of the constructs coding for the E1A deletion mutants was checked by Western blotting (Fig. 4A).

**Figure 5:**
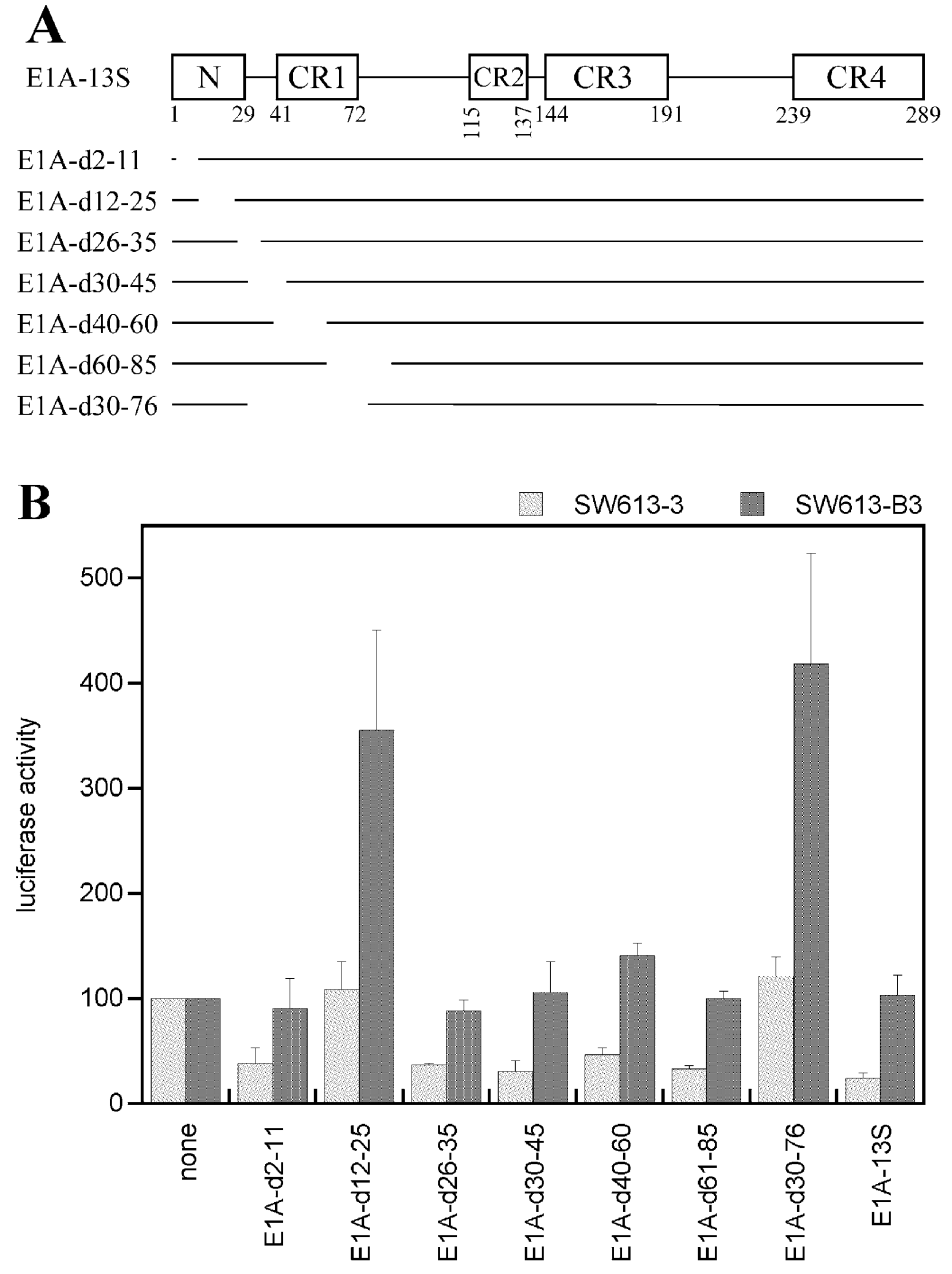
**Effect of internal deletion mutants of the E1A protein on the activity of the K18 promoter. (A) **Schematic representation of the structure of the mutants. Symbols are the same as in figure 3. **(B) **SW613-3 (T) and SW613-B3 (NT) cells were transfected with plasmid pK18(80)luc alone (none) or in the presence of the indicated E1A constructs. Results are presented as in figure 3. Error bars represent standard deviation of the mean of at least two experiments.

The results obtained with the N- and C-terminal deletion mutants, assigning a role to the first 25 aa in the inhibitory effect of E1A on the K18 promoter, were apparently contradictory to those obtained with deletion mutants E1A-12S-ΔCR1 and E1A-d30-76 (see above). To solve this issue, a series of small deletion mutants was constructed, spanning the region of aa 2 to 85 (Fig. 5A). With the exception of mutant E1A-d12-25, which has lost the inhibitory potential in T cells (and acquired a stimulatory effect in NT cells), all the other deletions in this region of the protein have no influence on the inhibitory effect of E1A and are unable to confer a stimulatory potential (Fig. 5B). In particular, mutants E1A-d26-35, -d30-45, -d40-60 and -d60-85, whose deletions span the region deleted in mutant E1A-12S-ΔCR1 and E1A-d30-76, have the same properties as wild type E1A with respect to the K18 promoter. The expression level of the E1A constructs used in the above-described experiments was checked by Western blotting (Fig. 4B). From all these results, we concluded that region 12–25 of E1A is involved in its inhibitory effect on the K18 promoter in T-type cells. The large deletion carried by mutants E1A-d30-76 and E1A-12S-ΔCR1 is adjacent to the 12–25 domain. A possible explanation for the results obtained with these mutants is that such large deletions may perturb the conformational structure of the molecule and functionally inactivates the 12–25 domain. It has been reported [25] that E1A mutants with deletions between residues 30–85 may not enter the nucleus, despite the presence of an unaltered nuclear localization signal at their C-terminus.

### A C-terminal domain of E1A is involved in the stimulatory effect on the K18 promoter

**Figure 6:**
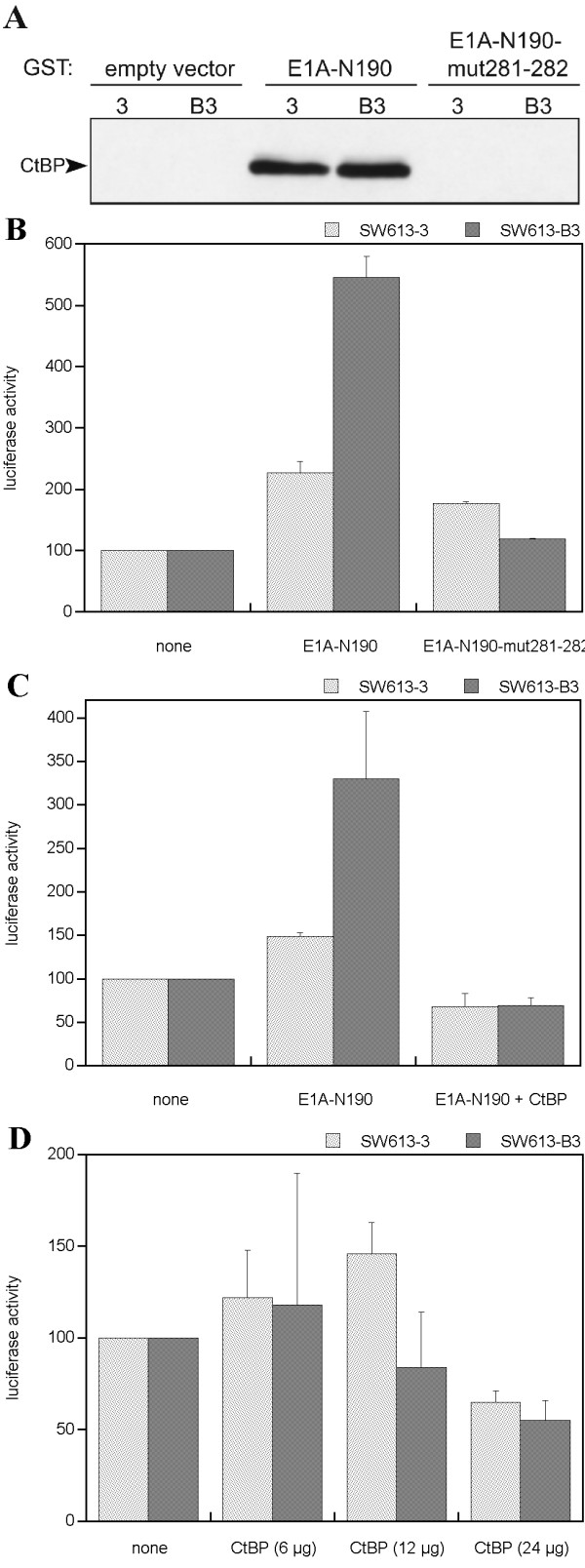
**CtBP1 has a role in the stimulatory effect of E1A mutants on the K18 promoter. (A) **CtBP1 interacts with E1A-N190 but not with the mutant E1A-N190-mut 281-282. GST pull-down assays were performed on extracts of SW613-3 (3) or SW613-B3 (B3) cells using GST alone (empty vector) or the fusion proteins GST-E1A-N190 or GST-E1A-N190-mut281-282. Bound proteins were analyzed by Western blotting with an anti-CtBP1 antibody. **(B) **Effect of the E1A-N190 or E1A-N190-mut281-282 proteins on the activity of the K18 promoter. SW613-3 (T) or SW613-B3 (NT) cells were transfected with plasmid pK18(80)luc alone (none) or in the presence of plasmid pE1A-N190 or pE1A-N190-mut281-282 as indicated. Results are presented as in figure 3. Error bars represent standard deviation of the mean of duplicate experimental points. **(C) **Reversion of the stimulatory effect of E1A-N190 by CtBP1. SW613-3 (T) or SW613-B3 (NT) cells were transfected with plasmid pK18(80)luc alone (none) or in the presence of plasmid pE1A-N190 (2 μg) or pcDNA3-CtBP (24 μg) or with the three plasmids together, as indicated. Results are presented as in figure 3. Error bars represent standard deviation of the mean of triplicate experimental points. **(D) **The CtBP1 protein has no direct effect on the activity of the K18 promoter. SW613-3 (T) or SW613-B3 (NT) cells were transfected with plasmid pK18(80)luc alone (none) or in the presence of various amounts of plasmid pcDNA3-CtBP, as indicated. Results are presented as in figure 3. Error bars represent standard deviation of the mean of triplicate experimental points.

**Figure 7:**
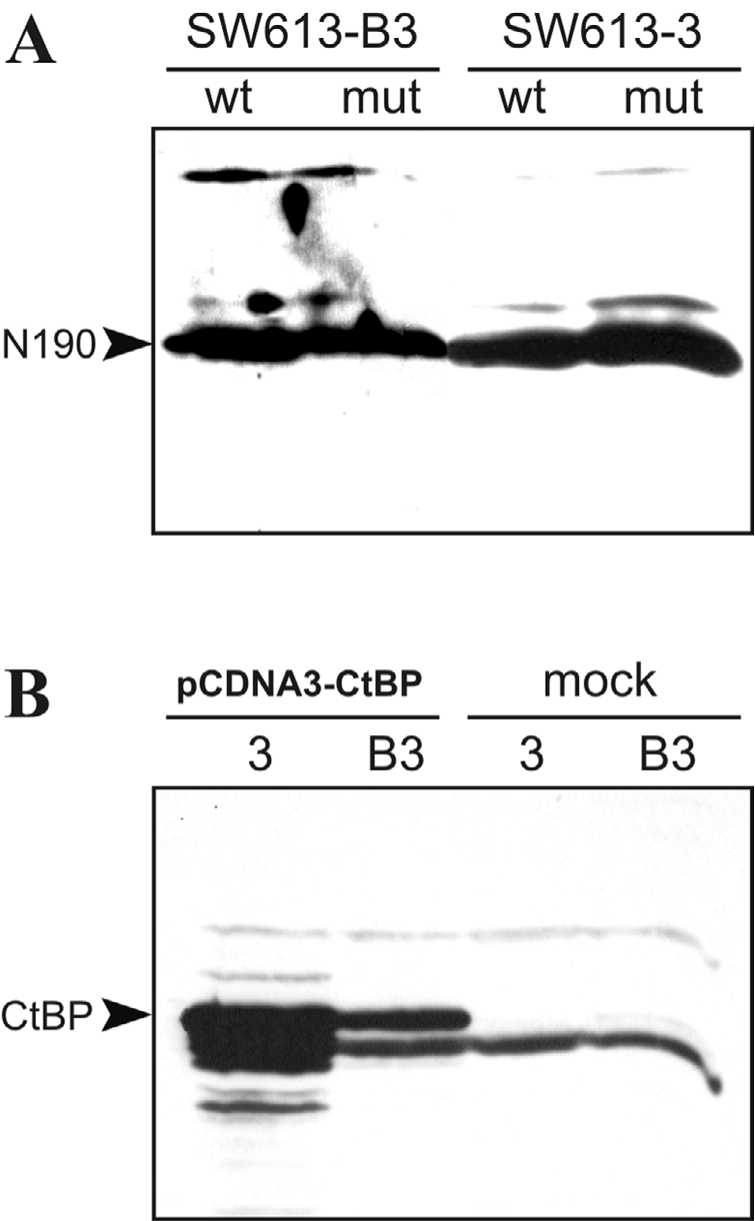
**Expression level of the E1A-N190 and CtBP1 constructs. (A) **SW613-B3 or SW613-3 cells were transfected with plasmid pE1A-N190 (wt) or pE1A-N190-mut281-282 (mut). Cellular extracts were analyzed by Western blotting with an anti-E1A antibody. **(B) **SW613-3 (3) or SW613-B3 (B3) cells were transfected with plasmid pcDNA3-CtBP or untransfected (mock). Cellular extracts were analyzed by Western blotting with an anti-CtBP1 antibody.

As mentioned above, all the N-terminal deletion mutants tested possess an activator effect on the K18 promoter, specifically in NT cells (Fig. 3C). Mutant E1A-N190 comprises only the last 99 aa of E1A-13S. On the other hand, none of the C-terminal mutants tested has acquired a stimulatory activity on the promoter in NT cells (Fig. 3B). From these results, we concluded that the region of E1A responsible for the stimulatory effect is located in the C-terminal part of the protein. The CtBP1 protein is known to interact with the PLDLS motif present in this C-terminal region of E1A [5]. To investigate a role for CtBP1 in the stimulatory effect of this region on the K18 promoter in NT cells, we used a previously characterized mutant of the PLDLS domain [6]. A two aa change (PLDLS→PLASS) destroys the capacity of binding to CtBP1 but does not affect the nuclear localization signal which is located nearby. We constructed the E1A-N190-mut281-282 mutant which is mutated on aa 281 (D→sA) and 282 (L→S). The binding of CtBP1 to E1A-N190 and the loss of interaction with E1A-N190-mut281-282 were confirmed by GST pull down assays and Western blotting (Fig. 6A). In transient expression assays (Fig. 6B), the E1A-N190-mut281-282 mutant cannot stimulate the K18 promoter in the NT cells. This result indicates that the binding to CtBP1 plays a role in the stimulatory effect of the C-terminal region of E1A on the activity of the K18 promoter in NT cells. This was confirmed by the observation that an excess of CtBP1 protein can abolish the stimulatory effect of E1A-N190 on the K18 promoter in NT cells (Fig. 6C), although CtBP1 by itself has no effect on the activity of the promoter (Fig. 6D). The expression level of the E1A-N190 and CtBP1 vectors was controlled by Western blot analysis (Fig. 7). A likely explanation for these results is that, in NT cells, the K18 promoter is repressed by a mechanism involving the binding of CtBP1 to the PLDLS motif of a partner protein. Stimulatory mutants such as E1A-N190 disrupt this interaction and relieve the repressed state of the promoter.

**Figure 8:**
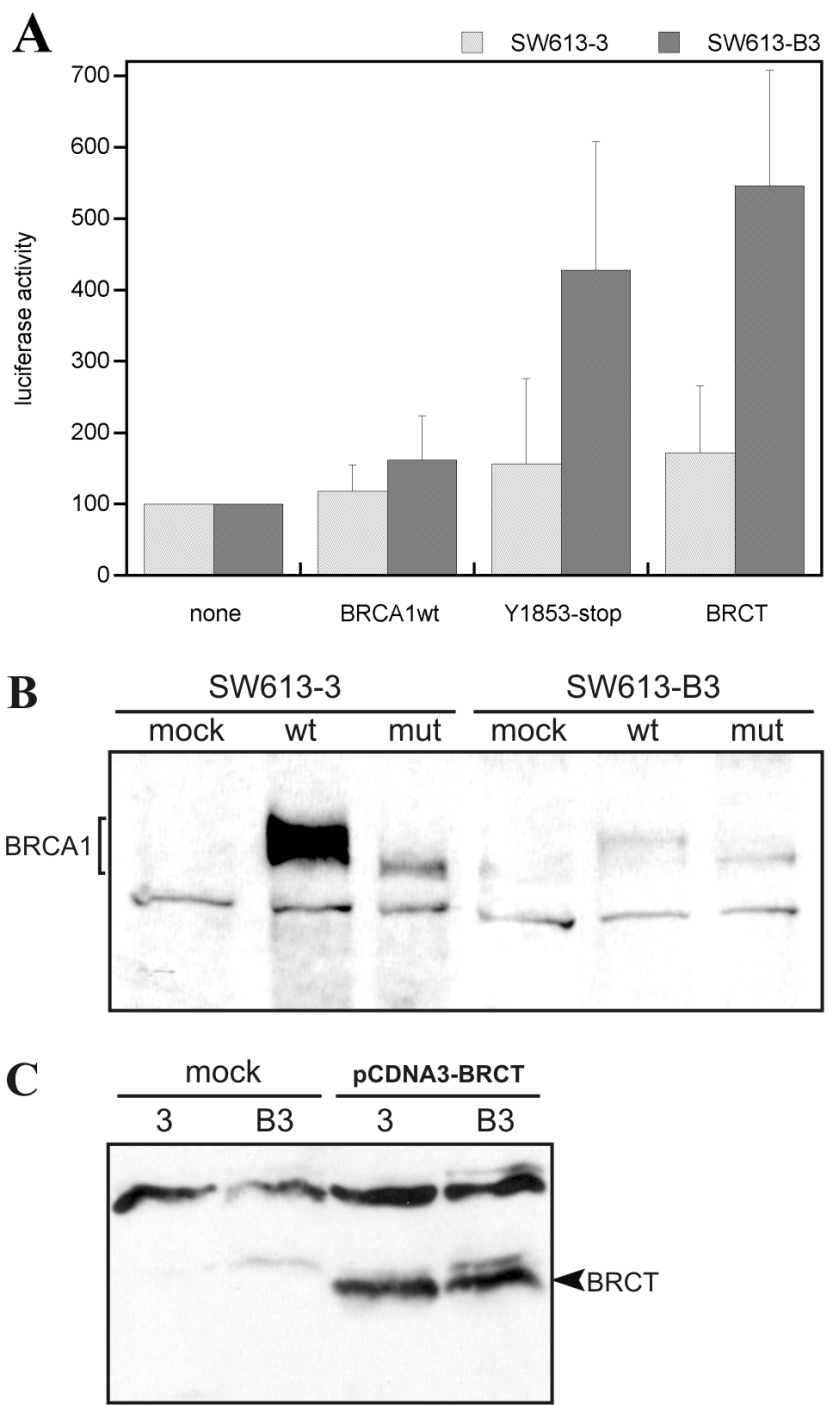
**The BRCA1 protein has a role in the repression of the K18 promoter in NT cells. (A) **SW613-3 (T) or SW613-B3 (NT) cells were transfected with plasmid pK18(80)luc alone (none) or in the presence of 24 μg of plasmids pCBRCA1C (BRCA1wt), pcDNA3-BRCA1(Y1853→STOP) or pcDNA3-BRCT, as indicated. Results are presented as in figure 3. Error bars represent standard deviation of the mean of three experiments. **(B) **and **(C) **Expression level of the BRCA1 constructs. SW613-3 (3) or SW613-B3 (B3) cells were untransfected (mock) or transfected with plasmid pCBRCA1C (wt), pcDNA3-BRCA1(Y1853→STOP) (mut) or pcDNA3-BRCT, as indicated. Cellular extracts were analyzed by Western blotting with an anti-HA antibody.

The differential activity of the K18 promoter and the inhibitory or stimulatory effect of various E1A mutant proteins are mediated by the minimal promoter ([22]; this work). No specific sequence of the promoter seems necessary for these effects (see above) which likely result from alterations in protein interactions in the preinitiation complex. It was reported that the CtBP1 protein is a corepressor which is recruited to promoters by DNA-bound transcription factors [4]. Therefore, our observations raise the question of how this protein is recruited to the preinitiation complex on the minimal K18 promoter. CtBP1 interacts with the carboxy-terminal interacting protein (CtIP) [6], which is a known partner of the Rb, LMO4 and BRCA1 proteins. A complex comprising LMO4, BRCA1 and CtIP was demonstrated in vivo [26]. The direct recruitment of the Rb protein to the transcription machinery has not been described to date but the presence of the BRCA1 protein in the RNA polymerase II holoenzyme has been reported [27,28]. This observation raises the possibility of a direct association of the BRCA1 protein with the preinitiation complex. The C-terminal BRCT repeats of BRCA1 are responsible for the interaction of this protein with CtIP [7,8]. The nonsense Y1853→STOP mutation identified in some familial breast cancers results in a 10-aa truncation of the second BRCT repeat and prevents the interaction of the mutant protein with CtIP. The possible effect of the wild type BRCA1 protein, of the BRCA1(Y1853→STOP) mutant and of a C-terminal fragment of the BRCA1 protein comprising only the BRCT repeats (pcDNA3-BRCT construct) on the activity of the K18 promoter was tested (Fig. 8A). If the BRCA1(Y1853→STOP) mutant has retained the capacity to be incorporated into the preinitiation complex, it should behave as a dominant negative mutant and stimulate the activity of the promoter, specifically in NT cells, since it is unable to recruit the CtIP-CtBP1 complex. If the BRCT fragment has lost the capacity to be incorporated into the preinitiation complex, it should also behave as a dominant negative mutant and stimulate the activity of the promoter, specifically in NT cells, since it should sequester the CtIP-CtBP1 complex away from the preinitiation complex. Wild type BRCA1 has no effect on the activity of the promoter in transient expression assays. The BRCA1(Y1853→STOP) mutant and the BRCT fragment do have a stimulatory effect on the activity of the K18 promoter, specifically in NT cells. The expression level of the BRCA1 constructs used in these experiments was assessed by Western blotting (Fig. 8B and 8C). The apparent dominant negative effect of the two BRCA1 mutants indicates that the interaction of BRCA1 with CtIP plays an important role in maintaining the repressed state of the K18 promoter in NT cells. Altogether, our results strongly suggest that the CtIP protein acts as an adapter molecule between BRCA1 and CtBP1, allowing the recruitment of CtBP1 to the preinitiation complex and the repression of the K18 promoter in NT cells.

## Discussion

### Differential activity of the K18 promoter and opposite effects of E1A mutants

We report here that the E1A protein isoforms (12S and 13S) and derivatives of E1A-13S that retain the N-terminal first 29 aa inhibit the activity of the K18 promoter, specifically in T-type cells of the SW613-S colon carcinoma cell line. In contrast, derivatives of the viral protein which have lost the inhibitory region but retain the C-terminal region of E1A stimulate the activity of the K18 promoter, specifically in NT cells. The two phenomena appear to be exclusive: among more than 30 E1A mutants studied (this work and unpublished results), none was found that could both repress the activity of the promoter in T cells and stimulate it in NT cells. This observation suggests that the two phenomena are related and could result from interference with the same and single mechanism. This is further supported by the observation that the stimulatory effect of mutant E1A-N25 is abolished in the presence of the wild-type E1A protein (data not shown). We have previously found that the differential activity of the K18 promoter between T and NT cells is a property of the minimal promoter (K18(41)) [21]. The mechanism responsible for this difference in activity does not involve the binding of a factor to a specific sequence of the promoter [22] but probably results from alterations of protein-protein interactions within the preinitiation complex. The same conclusion was reached here for the stimulatory or inhibitory effects of the various E1A derivatives on the activity of the K18 promoter. These effects were observed with the minimal promoter and with all the mutated versions of the K18 promoter tested. Altogether, these results strongly suggest that both inhibitory and stimulatory mutants of E1A exert their effect by interfering with the very mechanism responsible for the differential activity of the K18 promoter.

### A functional domain of the E1A protein located in the 12–25 region

We have found that a unique region of E1A, located between aa 12 and 25, is involved in its inhibitory effect on the activity of the K18 promoter. We previously reported results which suggested that E1A is acting on the p300 or CBP protein to inhibit the activity of the K18 promoter in T-type cells [23]. Two regions of the E1A protein, the N-terminal 1–28 region and the CR1 region, are known to be involved in the interaction of the viral protein with p300/CBP [29]. Within the CR1 region, the p300/CBP interacting domain is most probably located between aa 66 and 72. In the N-terminal region, two p300/CBP interacting domains have been identified. The first one spans residues 2–10 of E1A and the second domain is located between aa 19–28. It was shown [30,31] that aa 11–22 are not essential for the binding of E1A to p300/CBP. Thus, the 12–25 region involved in the inhibition of the K18 promoter appears to be different from the regions of interaction with the p300/CBP proteins. Furthermore, we have found that mutants E1A-ARG2 (R2G – data not shown), E1A-d2-11 and E1A-d61-85 which can no longer bind to p300/CBP, still efficiently inhibit the activity of the K18 promoter in T-type cells. Altogether, our results lead us to conclude that, contrary to what we suggested previously, inactivation of the p300/CBP proteins is most probably not involved in this inhibitory effect. It was reported that aa 1–21 and 55–60 of E1A are involved in its interaction with the PCAF factor [32,33]. The 1–21 region has not been further investigated so far to determine precisely the aa residues responsible for binding to PCAF. However, since the E1A-d40-60 mutant retains an inhibitory potential on the activity of the K18 promoter, this inhibition probably does not involve the PCAF protein. Within the first N-terminal 80 aa of E1A, the 12–26 region has the highest score for the probability of forming an α-helix and this property is conserved through five adenovirus serotypes [34]. The RAP30 subunit of TFIIF and the TATA-box binding protein (TBP) were shown to interact in vitro with aa 1–29 of E1A [35]. Severino et al [36] reported that aa 1–36 of E1A are responsible for the interaction with the RACK1 protein. We have screened a cDNA library by the yeast two-hybrid system using aa 12–29 of E1A as a bait. Clones corresponding to the glyceraldehyde-3-phosphate dehydrogenase (GAPDH), the UREB1 and the MYCBP proteins have been isolated (OB, ML, unpublished results). Experiments are in progress to determine if one of these factors is involved in the regulation of the K18 promoter in SW613-S cells.

### A role for CtBP1 and BRCA1 in the regulation of the K18 promoter

E1A mutants that have lost the 12–25 inhibitory region, acquired a stimulatory potential on the activity of the K18 promoter, specifically in NT cells, provided that they retain the C-terminal region of the viral protein. Within this region, aa 281–282, which are part of the CtBP1 binding site, are essential to the stimulatory effect and an excess of CtBP1 protein abolishes this effect. The CtBP1 protein is a corepressor, which represses promoters by HDAC-dependent or HDAC-independent mechanisms. We have previously shown [23] that the activity of the K18 promoter is stimulated by HDAC inhibitors (sodium butyrate or trichostatin), specifically in NT cells. We also made the observation that the K18 promoter could be stimulated in NT cells by forced recruitment to the promoter of a GAL4 fusion protein containing the histone acetyl-transferase (HAT) domain of CBP or of an acidic activator (GAL4-VP16) known to interact with HAT complexes. Mutations of aa residues involved in the HAT activity of CBP or in the interaction of VP16 with HAT complexes strongly reduced the stimulation. We conclude that the activity of the K18 promoter is repressed in NT cells by a mechanism involving the CtBP1 and HDAC proteins. CtBP1 is most probably acting through an HDAC-dependent mechanism because (i) we have reported [23] that the stimulatory effects by sodium butyrate and by the E1A-ΔCR1 mutant are not additive; (ii) forced recruitment of a GAL4-CtBP1 fusion protein on a K18 promoter engineered with GAL4 binding sites strongly inhibits the activity in both T and NT cells and this inhibition cannot be reversed by sodium butyrate (CD, unpublished results). These last results indicate that CtBP1 can inhibit the K18 promoter by an HDAC-independent mechanism but in this case, inhibition is not cell type specific. Stimulation of the activity of the K18 promoter by some E1A mutants in NT cells is most likely due to a relief of the repressive mechanism operating in these cells. The fact that these mutants have no stimulatory effect in T-type cells suggests that the high activity of the promoter reflects a reversal of the same repression mechanism, possibly through the expression of some constitutively high histone/factor acetyl-transferase (HAT/FAT) activity in these cells (see below). Our results are in agreement with those obtained by others. Grooteclaes et al. [37] reported that the K18 gene is derepressed in embryo fibroblasts from mice with homozygous compound knockout of the CtBP1 and CtBP2 genes. In addition, the K18 gene is repressed in knockout cells infected with a CtBP1 retroviral vector. Schuierer et al. [38] found that the E1A protein activates the expression of the AP2α gene by interfering with CtBP1. Sundqvist et al. [39] have reported that the CtBP1 interacting region of E1A relieves the HDAC-dependent repression of transcription by CtBP1.

We propose that CtBP1 and associated HDAC could be recruited to the preinitiation complex by a BRCA1/CtIP complex. Such a role for BRCA1 was not reported previously although it is known that BRCA1 is associated with the RNA polymerase II holoenzyme. Some examples of transcriptional repression by BRCA1 were reported. It represses c-MYC-mediated stimulation of the human telomerase reverse transcriptase promoter [40]. BRCA1 functions as a corepressor for the transcriptional repressor ZBRK1 [41]. Experiments using DNA microarrays have indicated that the BRCA1, CtBP1, CtBP2 and CtIP genes are expressed at comparable levels in T and NT cells of the SW613-S cell line (C. Lavialle, personal communication). This was also the case for genes coding for HDAC-1 to -4 and HDAC-6 to -10. The HDAC-5 gene was found to be overexpressed 3.4 fold in NT cells, as compared to T cells, and this was confirmed by Western blot analysis. However, in transient expression assays, the activity of the K18 promoter was unaffected by overproducing the HDAC-5 protein, both in T and NT cells (CD, unpublished results), indicating that the low level of accumulation of HDAC-5 in T-type cells is not responsible for the high activity of the promoter in these cells. It could be argue that a gene coding for one of these factors is specifically mutated in T cells. This seems highly unlikely since the K18 promoter is also deregulated in stable transfectants obtained from NT cells using a c-MYC expression vector and which acquired all the phenotypic properties of T-type cells (PP, unpublished results).

### A model for the differential activity of the K18 promoter

**Figure 9:**
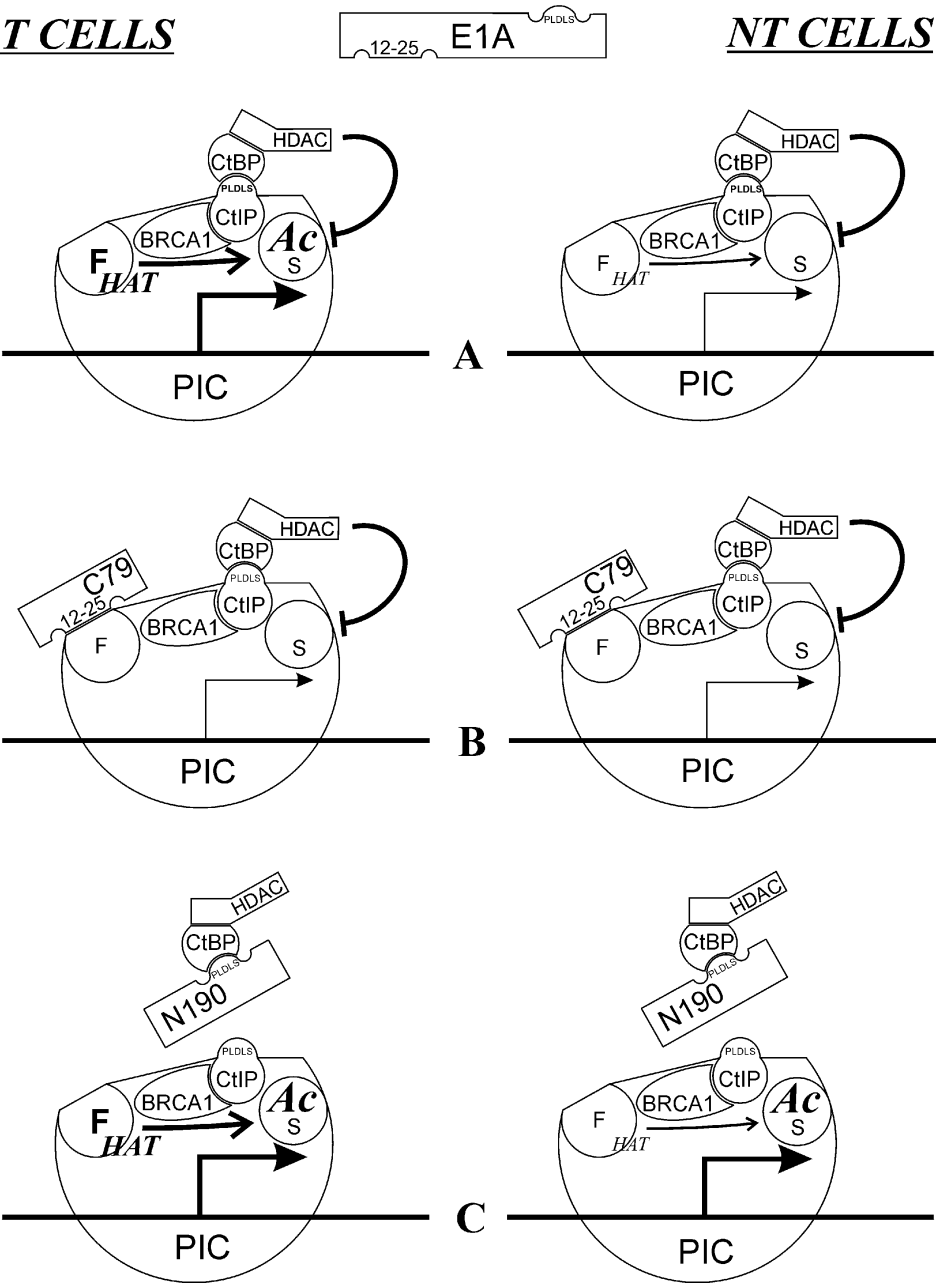
**A model for the putative mechanism responsible for the differential activity of the K18 promoter and for the effect of the different E1A mutants**. The E1A full-length protein is represented at the top with emphasis on the two domains of interest: aa 12 to 25 and the PLDLS motif. The horizontal line represents the DNA and the initiation site is symbolized by an arrow, whose thickness is an indication of the activity of the promoter. **(A) **Functioning of the K18 promoter in T and NT cells. The F factor is an as yet unidentified protein which has a HAT/FAT activity and is supposedly more abundant or more active in T cells (bold) than in NT cells. F acetylates (Ac) the substrate protein S, a non-histone protein whose acetylation level controls the activity of the K18 promoter. The acetylation level of S is also under the control of HDAC proteins. The CtBP1 protein interacts with the PLDLS motif of the CtIP protein. CtBP1 associates with some HDAC proteins by a domain that is different from the one recognizing the PLDLS motif [4, 48]. The recruited HDAC proteins deacetylate the S substrate in NT cells where the acetylase activity is already weak and this down-regulates the promoter. CtIP is an interaction partner of BRCA1 which is represented here as part of the preinitiation complex (PIC). **(B) **Effects of C-terminal deletion mutants of E1A on the activity of the K18 promoter in T and NT cells. The binding of these mutants to the F factor through the 12–25 domain results in a low HAT/FAT activity, deacetylation of S and inhibition of the promoter in T cells. These mutants are expected to have little effect in NT cells since the level of acetylation of S is already low in these cells. **(C) **Effects of N-terminal deletion mutants of E1A on the activity of the K18 promoter. The C-terminal part of E1A contains the PLDLS motif which permits its interaction with CtBP1. The displacement of CtBP1 from CtIP prevents the recruitment of HDAC proteins to the preinitiation complex. This is of no consequence in T cells where the S protein is maintained in an hyperacetylated state by the high HAT/FAT activity. In contrast, in NT cells removal of HDAC proteins allows the weak HAT/FAT activity to acetylate S to a high level which results in a stimulation of the promoter.

To summarize the observations we made on the differential regulation of the K18 promoter between T and NT cells of the SW613-S cell line, we propose the model presented in figure 9. Several aspects are still speculative but it is compatible with all the results we have obtained so far. The expression level of the K18 gene in NT cells is comparable to that observed in normal epithelial cells of human colon [42]. Our results indicate that, in NT cells, the K18 promoter is in fact subjected to a repression mechanism involving the CtBP1 protein and HDACs, probably in order to keep in check the expression level of the K18 gene. The data presented suggest that CtBP1 and associated HDACs could be recruited to the preinitiation complex by a BRCA1/CtIP complex (Fig. 9A). In our model, the target of HDACs is a factor (S), whose acetylation level is controlled by a balance between HDAC and HAT/FAT activities. The activity of the K18 promoter would be dependent on the acetylation level of S. We have reported previously that the acetylation state of histones H3 and H4, as well as the structure of the chromatin, are the same in T and NT cells in the region of the K18 promoter [23]. In addition, the mechanism responsible for the differential activity is acting on the minimal promoter and no specific sequence is required. Therefore, we propose that S is a non-histone protein which is a component of the preinitiation complex and is acetylated by a FAT activity.

In T-type cells, the FAT activity recruited to the preinitiation complex would be higher, because the enzyme is overproduced or more active in these cells. The high FAT activity would shift the balance toward an hyperacetylated state of S. We propose that this protein with FAT activity (F) is the target of the 12–25 domain of E1A (Fig. 9B). The viral protein, or its mutant forms retaining a functional 12–25 domain, would inhibit the FAT activity. This would result in the inhibition of the promoter. It is very likely that the factor with FAT activity is not the p300/CBP or PCAF proteins because we identified E1A mutants disabled in their capacity to bind to these factors but that are still able to efficiently inhibit the activity of the promoter in T-type cells. In the context of our model and as a novel candidate FAT activities, it is interesting to note recent reports describing the autoacetylation of the general transcription factor TFIIB [43] and of the RAP30 subunit of TFIIF [44].

E1A mutants with no functional 12–25 domain but retaining the C-terminal PLDLS motif would prevent by competition the recruitment of CtBP1 and HDACs to the preinitiation complex (Fig. 9C). In NT cells, this would shift the balance towards hyperacetylation of S despite the low FAT activity of the F protein, resulting in a stimulation of the promoter. Such mutants are not expected to have an effect in T-type cells since the high FAT activity in these cells already supersede that of HDACs. Finally, the proposed model offers an explanation to the observation that inhibition of the promoter in T cells and stimulation in NT cells are apparently exclusive phenomena. Indeed, according to the model, no E1A mutant is expected to have both capabilities.

## Conclusion

The promoter of the keratin 18 gene is deregulated in cells of the human colon carcinoma cell line SW613-S by an unusual mechanism that is acting on the minimal promoter and involves alteration of an acetylation mechanism acting on a non-histone substrate. We report here that the adenoviral E1A protein and some of its mutants specifically interfere with this mechanism through two regions of the protein: the C-terminal CtBP1 binding domain and a domain spanning aa 12–25. Our results lead us to conclude that, in colon epithelial cells, the expression level of the K18 gene is kept in check by a repression mechanism involving the CtBP1, HDAC and BRCA1 proteins. This mechanism is altered in SW613-S colon carcinoma cells that overexpress the K18 gene. Since it is acting at the level of the preinitiation complex, its alteration most probably participates in the deregulated expression of many genes in these tumor cells.

## Methods

### Cell lines, transfection and luciferase assays

The origin of the SW613-S cell line and cell culture conditions have been described previously [10,16]. Clones SW613-3 (T-type cells) and SW613-B3 (NT-type cells) were used in this study. Transfection and luciferase assays were performed in triplicate for each construct in each experiment, as described previously [21,22] and six micrograms of each plasmid were used, unless otherwise stated. In cotransfection experiments where the reporter plasmid was co-introduced with expression vectors coding for various polypeptides, the reference assay comprised cells transfected with the reporter plasmid and with an amount of empty vector corresponding to the molar equivalent of the expression vector used. In each experiment, the two cell types were also transfected in parallel with the pSVluc construct and the activity of every promoter construct was expressed relative to that of the SV40 early promoter. We have shown previously that this viral promoter is equally active in both cell types [21]. This is also the case for the human cytomegalovirus IE1 gene promoter which drives the expression of the various polypeptides encoded by the vectors used in co-transfection experiments (CD, unpublished observations).

### Plasmids constructions

Construction of plasmids pK18(41)luc, pK18(80)luc and pSVluc has already been reported [22]. Plasmids pE1A-13S, pE1A-C249, -C192, -C139, -C109, -C79, -N25, -N76, -N120, -N132 [33] were given to us by Dr R.M. Evans (Salk Institute for Biological sciences, La Jolla, USA). Plasmids pE1A-d2-11 and pE1A-d26-35 [45] were gifts of Dr. M. Fuchs (Dana-Farber Cancer Institute, Boston, USA). Plasmids pcDNA3-CtBP and pGEX-CtBP [6,39] coding for the human CtBP1 protein were given to us by Dr. C. Svensson (Uppsala University, Sweden). Plasmids pCBRCA1C (coding for wild type BRCA1) and pcDNA3-BRCA1(Y1853→STOP) were provided by Dr J. Feunteun (Institute Gustave Roussy, Villejuif, France). A fragment coding aa 1640 to 1864 of BRCA1 was amplified by the polymerase chain reaction (PCR) from plasmid pGEX4T1-BRCT [46], a gift from Dr N. Dalla Venezia (Université Médicale Rockefeller, Lyon, France), and inserted into the vector pcDNA3. A DNA fragment coding for 3 tag HA motifs was then inserted upstream of the BRCT coding region to yield plasmid pcDNA3-BRCT. Plasmid pE1A-d30-76 was obtained by inserting into plasmid pE1A-N76 a fragment amplified by PCR from plasmid pE1A-13S and coding for aa 1 to 29 of E1A. For all E1A constructs, aa numbering refers to the E1A-13S isoform. Plasmids pE1Ad30-45, pE1A-d40-60, pE1A-d61-85 were constructed by inserting into the pCMX-PL1 vector two fragments coding respectively for aa 1–29 and 46–289, 1–39 and 61–289 or 1–60 and 86–289 of E1A-13S. Plasmid pE1A-d12-25 was obtained by inserting into plasmid pE1A-N25 a double-stranded synthetic oligonucleotide coding for aa 1 to 11. Plasmid pE1A-C29 was derived from plasmid pE1A-d30-76 by deleting the sequence corresponding to aa 77 to 289. Plasmids pE1A-N190 and pE1A-N190-mut281-282 were constructed by inserting into the pCMX-PL1 vector a fragment amplified by PCR coding respectively for the wild type sequence of aa 190 to 289 or a sequence with two mis-sense mutations on aa 281 (D→A) and 282 (L→S). The same PCR fragments were also inserted into plasmid pGEX-1LambdaT coding the glutathion-S-transferase (GST) protein to obtain plasmids pGEX-E1A-N190 and pGEX-E1A-N190-mut281-282. All constructs coding for E1A proteins were checked by sequencing.

### GST-pull down assays

A 40 ml overnight culture of bacteria transformed with the appropriate plasmid was diluted 10-fold with culture medium and incubated for one hour at 37°C with shaking. Induction was for 3 hours in the presence of isopropylthiogalactoside (1 mM). The bacteria were pelleted by centrifugation (10 min, 4000 rpm), resuspended in 20 ml of phosphate-buffered saline (PBS) containing Complete Inhibitor EDTA free (Roche) and 1 mM phenyl-methylsulfonyl fluoride (PMSF) (Sigma) and disrupted by sonication (twice 15 s, 50% of full power). The suspension was adjusted to 1% of Triton X-100 and incubated for 30 min at 4°C. After centrifugation (30 min, 13 000 rpm, 4°C), the supernatant was collected and stored at -80°C as one ml aliquots. The concentration of the GST-fusion protein was determined by purifying it from a one ml aliquot using glutathione beads. Quantification was carried out by migration on a polyacrylamide gel, using a range of known quantities of bovine serum albumin as a standard. For the pull-down assays, 10 μg of GST fusion proteins and 50 μl of a 50% slurry of glutathione Sepharose 4B beads (Pharmacia Biotech) were mixed and diluted to 1 ml with PBS. Incubation was for 2 hours at 4°C with gentle shaking. Beads were washed twice with PBS and once with the protein extraction buffer (50 mM Tris-HCl pH 7.5, 150 mM NaCl, 1% NP40 and 1 mM EDTA). The beads were incubated with cellular protein extracts for 2 hours at 4°C, washed with protein extraction buffer and boiled in loading buffer.

### Western blotting

Samples of cellular extracts (300 μg of proteins) or from the pull-down assays were analyzed by migration on 15% SDS-polyacrylamide gels (6% for the BRCA1 and BRCA1(Y1853→STOP) proteins). Western blotting onto nitrocellulose membranes was performed using a semi-dry transfer procedure [47] or a liquid transfer method (400 mA overnight in a Tris-base 25 mM, glycin 200 mM solution) in the case of BRCA1. Blocking of the membrane and incubation with the antibody were performed in a solution containing 10 mM Tris-HCl pH 8.0, 150 mM NaCl, 0.05% Tween 20 and 5% dry low-fat milk. The mouse monoclonal anti-HA antibody was a gift from Dr. S. Leibovitch (Institute Gustave Roussy, Villejuif, France). Anti-E1A (SC430) and anti-CtBP1 (SC11390) rabbit polyclonal antibodies were obtained from Santa-Cruz Biotechnology, Inc.

## Authors' contributions

CD carried out the protein biochemistry studies, sequence analysis and part of the molecular genetic studies and drafted the manuscript. PP carried out part of the molecular genetic studies and helped to draft the manuscript. ML carried out part of the molecular genetic studies. OB conceived the study, participated in its design, coordinated it and helped to draft the manuscript.
